# Enhanced Detection of BRCA Copy Number Alterations Within a Commercial HRD Assay: Implications for Precision Oncology in Ovarian Cancer

**DOI:** 10.3390/ijms27093965

**Published:** 2026-04-29

**Authors:** Maria De Bonis, Pierluigi Iapicca, Elisa De Paolis, Francesca Brisighelli, Jessica Evangelista, Alessia Perrucci, Claudio Ricciardi Tenore, Giulia Maneri, Paola Concolino, Alessia Piermattei, Iolanda Mozzetta, Tina Pasciuto, Alessia Preziosi, Luciano Giacò, Simona Duranti, Camilla Nero, Anna Fagotti, Angelo Minucci

**Affiliations:** 1 Departmental Unit of Molecular and Genomic Diagnostics, Fondazione Policlinico Gemelli IRCCS, 00168 Rome, Italy; maria.debonis@policlinicogemelli.it (M.D.B.);; 2 Genomics Core Facility, G-STeP, Fondazione Policlinico Universitario Agostino Gemelli IRCCS, 00168 Rome, Italy; 3 SOPHiA GENETICS, 1180 Rolle, Switzerland; 4 Pathology Unit, Department of Woman and Child’s Health and Public Health Sciences, Fondazione Policlinico Universitario Agostino Gemelli IRCCS, 00168 Rome, Italy; 5 Research Core Facility Data Collection G-STeP, Fondazione Policlinico Universitario Agostino Gemelli IRCCS, 00168 Rome, Italy; 6 Bioinformatics Research Core Facility, Gemelli Science and Technology Park (G-STeP), Fondazione Policlinico Universitario Agostino Gemelli IRCCS, 00168 Rome, Italy; 7 Unit of Oncological Gynecology, Department of Women, Children and Public Health Sciences, Fondazione Policlinico Universitario Agostino Gemelli IRCCS, 00168 Rome, Italy; 8 Department of Women, Children and Public Health Sciences Università Cattolica del Sacro Cuore, 00168 Rome, Italy

**Keywords:** NGS, ovarian cancer, *BRCA1/2*, HRD, CNA, LGRs, genomic instability

## Abstract

Large genomic rearrangements (LGRs), occurring as copy number alterations (CNAs), represent a clinically relevant class of pathogenic or likely pathogenic variants (P LPVs) in *BRCA1/2* (*BRCA*) genes in ovarian cancer (OC). We evaluated the performance of a high-resolution algorithm integrated into a commercial homologous recombination deficiency (HRD) assay to improve the identification of clinically actionable CNAs in *BRCA* genes by formalin-fixed paraffin-embedded (FFPE) samples. A total of 760 OC samples were analyzed using a commercial HRD assay incorporating a bioinformatics algorithm for CNA detection. The algorithm was additionally applied to additional homologous recombination repair (HRR) genes, and associations between CNA events and genomic instability (GI) were evaluated. The algorithm demonstrated high sensitivity for both gene and exon-level CNA. The high correlation between CNA positivity cases and GI, in the absence of P/LPVs *BRCA* single-nucleotide or *indels* variants, emphasizes the value of integrating CNA detection into routine HRD testing workflows. The extended analysis of additional HRR genes enabled broader characterization of clinically relevant CNAs. This study enables reliable identification of clinically relevant *BRCA* LGRs from FFPE within HRD testing, supporting a tumor-first diagnostic strategy. This approach may expand the identification of OC patients potentially eligible for PARP inhibitor therapy.

## 1. Introduction

Ovarian cancer (OC) remains one of the most lethal gynecologic malignancies, with BRCA1/2 (BRCA) pathogenic/likely pathogenic variants (P/LPVs) significantly influencing prognosis and treatment decisions. While small-scale BRCA variants, such as single-nucleotide variants (SNVs) and insertions/deletions (indels), are routinely detected with high sensitivity and specificity using next-generation sequencing (NGS), reliable detection of large genomic rearrangements (LGRs), typically manifesting as copy number alterations (CNAs), remains technically challenging, particularly in tumor tissue samples [1,2,3].

LGRs in *BRCA* can affect gene function and have a direct impact on therapeutic choices and response, particularly in the context of poly (ADP-ribose) polymerase inhibitors (PARPi) treatments. As such, their reliable identification is recognized as an essential component of genomic profiling in OC [4,5].

Homologous recombination deficiency (HRD) assays have a central role in the molecular characterization of OC, enabling clinicians to identify patients who are more likely to benefit from DNA-damaging-based targeted therapies. However, many currently available HRD assays offer limited resolution for detecting exon-level CNAs, particularly in somatic contexts where tumor heterogeneity and formalin fixation may compromise data quality. This limitation is compounded by the absence of a validated gold standard for somatic CNAs detection, which has contributed to inconsistencies across platforms and uncertainty in clinical interpretation [6,7].

Recent advances in computational approaches have enabled the development of increasingly refined CNA detection algorithms, capable of integrating read depth, allelic imbalance, and fragment distribution to identify exon-level rearrangements with improved sensitivity and specificity. Several established HRD assays, including Myriad myChoice^®^ CDx and FoundationOne^®^ CDx, incorporate CNA or LGR detection within their analytical frameworks. However, these platforms are based on centralized testing models that typically require sample referral to dedicated laboratories.

In parallel, decentralized, laboratory-implementable HRD solutions are emerging, offering the possibility to integrate genomic profiling directly into routine diagnostic workflows. The incorporation of CNA detection within in-house HRD testing may improve diagnostic completeness by enabling the identification of clinically relevant quantitative alterations within a single assay [8,9].

Within this context, we evaluated the performance of a CNA analysis module integrated into a commercially available, laboratory-implementable HRD assay for the detection of exon-level LGRs in BRCA genes from FFPE tumor samples. The focus of this study is the clinical evaluation of CNA detection performance in a large real-world ovarian cancer cohort. The analysis was subsequently extended to 19 additional HRR genes, enabling a broader genomic characterization within the same workflow.

Overall, this study provides a large-scale assessment of CNA detection in a decentralized HRD testing setting, including comparison with orthogonal methodologies and evaluation of its applicability in routine diagnostic practice and tumor-first testing strategies in OC patients.

## 2. Results

### 2.1. Performance of BRCA CNA Detection

A total of 760 patients were analyzed to assess the performance of the CNA detection methodology in the *BRCA1/2* genes. One sample was subsequently excluded during the QC process due to insufficient data quality, resulting in 759 evaluable patients. The median age was 62 years (range, 23–88). High-grade serous carcinoma accounted for ~90% of cases, endometrioid carcinoma ~5%, and the remaining ~5% included low-grade serous, mucinous, clear cell, undifferentiated/dedifferentiated carcinoma, carcinosarcoma, malignant Brenner tumor, ovarian metastases, and mixed histologies.

A total of 1592 events were recorded and assessed across all samples, regardless of whether they involved *BRCA1* or *BRCA2*, including multiple events from the same sample. Of these, 28 events (1.8%) were excluded due to quality control checks or other rejection criteria, yielding 1564 evaluable events. Among these, 1509 events (96.5%) were classified as normal, indicating no CNV in *BRCA1* or *BRCA2* (Table 1), while 55 events (3.5%) were identified as CNA-positive, corresponding to either gain or loss events in *BRCA1* and/or *BRCA2*.

**Figure 1:**
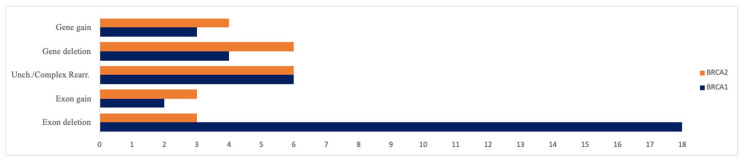
Types of CNV events identified in *BRCA1* and *BRCA2*. Schematic representation of the copy number variation (CNV) events detected in *BRCA1* and *BRCA2* genes, including whole-gene deletions, multi-exonic deletions, single-exon losses, and exon-level duplications. The figure illustrates the distribution and relative frequency of CNV types identified across the analyzed ovarian cancer cohort using the HRD assay integrated with the novel CNV detection algorithm.

Among the CNA-positive calls, the most frequent alteration was exon loss (single or multiple exons) at 38%, followed by complex or uncharacterized rearrangements at 22%. Complete gene loss was observed in 18% of events, complete gene gain in 13%, and exon/multi-exon gain in 9% of cases (Figure 1).

Across the 759 evaluable samples, 95% (721/759) were classified as normal/wild type (WT), while 5% (38/759) harbored a P/LP CNA (Table 2 and Table 3).

**Figure 2:**
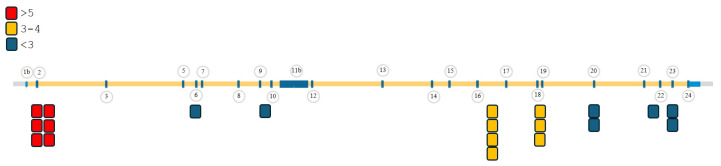
Exon-level CNV events identified in the *BRCA1* gene. Distribution of exon-level CNVs detected in the BRCA1 gene across the analyzed ovarian cancer cohort. Exon 2 (*n* = 6) and Exons 16–17 (*n* = 4) were the most frequently affected regions, as identified using the HRD assay integrated with the novel CNV detection algorithm.

Of the pathogenic CNAs identified, 68% (26/38) affected *BRCA1* and 32% (12/38) affected *BRCA2*, consistent with previously reported proportions in hereditary ovarian and breast cancer datasets (*BRCA1*: 66%; *BRCA2*: 34%) [10]. The most frequently affected exons were Exon 2 (*n* = 6) and Exons 16–17 (*n* = 4) of *BRCA1* (Figure 2).

Interestingly, in a subset of samples with known GI status (Table 2), a positive GI result was associated with pathogenic CNA positivity in 77% of cases (26/34). Notably, more than 70% of cases (19/26) retained *BRCA* WT sequences, with no pathogenic SNVs or *indels* detected. In line with this observation, all GI-negative cases displayed a WT status for both *BRCA1* and *BRCA2* (8/8), as summarized in Table 4.

### 2.2. CNA Detection Performance Compared to Orthogonal Assays

Orthogonal testing data were available for a subset of cases (*n* = 73), assessed using alternative assays, including the amplicon-based assay (Devyser BRCA) and Multiplex Ligation-dependent Probe Amplification (MLPA) (MRC Holland, Amsterdam, North Holland, Netherlands). In this subset, the HRD assay CNA tool (SOPHiA DDM software version 4, Sophia Genetics SA, Rolle, Switzerland) demonstrated high concordance, correctly identifying CNAs in 96% of cases (70/73). After excluding two cases for which the CNA tool did not generate a result, concordance increased to 99%. A single discrepant case required further investigation: MLPA testing excluded a germline of origin, leaving the somatic status unresolved and suggesting a potential false positive.

As with SNVs, tumor-only testing cannot definitively distinguish somatic from germline whole-gene deletions. Therefore, confirmatory germline testing is recommended for any CNA identified. In a subset of 17 CNA-positive samples subjected to parallel germline CNA analysis, 40% (7/17) showed a WT profile, confirming the somatic origin of these events and underscoring the importance of assessing *BRCA* CNAs in tumor tissue.

Analysis of blood-derived samples further supports the assay’s reliability. All 10 CNA calls in blood were confirmed as germline by MLPA, while 55/55 WT blood samples showed no CNAs, confirming concordance and demonstrating a very low risk of false-positive calls in germline DNA. These findings highlight the robustness of the CNA tool in accurately detecting both somatic and germline events.

### 2.3. Frequency of Gene-Loss and Exon-Level Events in HRR Genes

**Figure 3:**
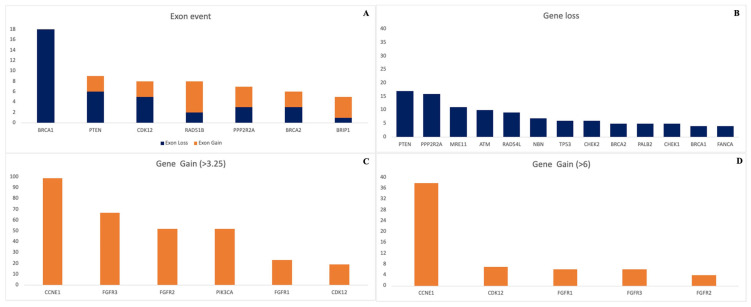
Overview of CNAs detected in ovarian cancer samples. (**A**) Exon-level events showing *BRCA1* as the most frequent exon loss, followed by *PTEN* and *CDK12*; sensitivity may be reduced for smaller genes. (**B**) Gene losses most common in *PTEN*, *PPP2R2A*, *MRE11*, and *ATM*. (**C**) Gene gains (>3.25 copies) predominantly in *CCNE1*, with amplifications also in *FGFR3*, *FGFR2*, *PIK3CA*, *FGFR1*, and *CDK12*. (**D**) High-level gene gains (>6 copies) mainly observed in *CCNE1*, showing limited overlap with HRD-positive cases.

The CNA algorithm provides gene-level and exon-level resolution for 19 additional genes involved in HRR, as covered by the HRD assay. Figure 3 summarizes the CNA outcomes reported by the tool. Among HRR genes, *BRCA1* was the most frequently affected by exon-loss events, followed by *PTEN* and *CDK12* genes. It should be noted that exon-level resolution may be influenced by gene size; thus, detection sensitivity for smaller genes, such as *TP53*, *RAD51D*, *PTEN*, and *RAD51C*, may be reduced compared to larger genes in the panel.

At the gene-loss level, the most frequently affected genes were *PTEN*, *PPP2R2A*, *MRE11*, and *ATM*, each exhibiting more than 10 events. The most prevalent gene gain was observed in *CCNE1*, occurring in 13% of patients, consistent with previous reports [11,12,13,14], followed by *FGFR3* (9%), *FGFR2* and *PIK3CA* (7%), and *FGFR1* and *CDK12* (3%).

Given the emerging therapeutic relevance of *CCNE1*, we assessed potential correlations between *CCNE1* amplification status and HRD score. As summarized in Table 5 and in agreement with other datasets [12], high-level CCNE1 amplification (>6 copies, as defined by the CNA tool) rarely correlated with an HRD-positive score.

## 3. Discussion

In this study, we evaluated the performance of a CNA detection module integrated into a commercially available, laboratory-implementable HRD assay for the identification of BRCA CNAs in OC. Our findings highlight the robustness of the CNA detection component within a real-world clinical setting, supporting its role as an integral part of comprehensive HRD assessment. Established platforms, such as Myriad myChoice^®^ CDx (Myriad Genetics, Salt Lake City, UT, USA) and FoundationOne^®^ CDx (Foundation Medicine, Inc., Cambridge, MA, USA), also include CNA or LGR detection within centralized testing frameworks widely used in clinical practice. However, the present work specifically explores the performance of a CNA module embedded in a decentralized, laboratory-implementable workflow, where CNA analysis can be performed in parallel with HRD scoring within a single diagnostic process. Importantly, this integrated approach allows for a more complete genomic characterization of BRCA alterations without the need for additional reflex testing.

Large genomic rearrangements (LGRs) manifested as CNAs in *BRCA* genes represent a distinct class of P/LPVs with critical clinical implications. International guidelines, including those from the CCMG and NHS UK, recommend the systematic assessment of these variants, as their functional consequences depend on their size and genomic location, potentially resulting in frameshifts, nonsense-mediated decay, or loss of essential protein domains [1,2,3,4,10,11,15].

*BRCA* testing in OC patients is primarily performed on FFPE samples, reflecting a tumor-first diagnostic strategy that is increasingly adopted in routine clinical oncology practice. With the introduction of HRD testing, *BRCA* analysis has now been incorporated into this assay. Consequently, there is a growing need to optimize HRD testing to ensure the comprehensive detection of all possible *BRCA* alterations, including CNAs.

In this study, we evaluated the performance of a CNA detection module integrated into a commercially available, laboratory-implementable HRD assay within our institutional diagnostic workflow. Across 760 samples, the module demonstrated robust analytical performance, with 98.2% of assessed events passing quality control. The frequency of CNA-positive events observed in our cohort is consistent with previously reported estimates of BRCA LGRs prevalence in ovarian cancer cohorts [16,17,18].

Exon-level deletions represented the most frequent type of CNA detected in our cohort, predominantly affecting the *BRCA1* gene. This finding underscores the importance of adopting high-resolution CNA detection approaches, as lower-resolution assays may fail to identify potentially clinically actionable intragenic alterations and may therefore require additional testing to ensure their detection. Despite technical challenges related to FFPE samples, such as DNA fragmentation, coverage variability, and tumor heterogeneity, the assay maintained strong performance. This highlights the robustness of the CNA tool even in the highly unstable genomes characteristic of the OC, which exhibits a high burden of somatic CNAs relative to SNVs [4,17,18].

Comparison with orthogonal methodologies (MLPA and amplicon-based NGS assays) showed excellent concordance, reaching 99% when non-informative cases were excluded. Although tumor-only testing cannot definitively distinguish somatic from germline CNAs, our data demonstrate that germline CNAs can be reliably detected from tumor-derived sequencing data. All CNA calls in blood samples were confirmed by MLPA, and CNAs identified in tumor tissue that were subsequently shown to be germline were consistently detected by the assay [4,19].

These findings support a tumor-first testing workflow, in which somatic CNA analysis serves as an effective initial screening step to identify clinically relevant *BRCA* rearrangements. Variants detected in tumor tissue can then be confirmed through reflex germline testing and, if appropriate, integrated into genetic counseling and patient management strategies. This approach optimizes both diagnostic efficiency and resource allocation while ensuring accurate identification of clinically actionable CNAs [4,20,21].

Beyond *BRCA*, the CNA algorithm enabled gene- and exon-level interrogation of 19 additional HRR-related genes directly from tumor tissue. Recurrent gene losses were observed in *PTEN*, *CDK12*, *PPP2R2A*, and *MRE11*, which are increasingly recognized for their role in homologous recombination deficiency and responsiveness to DNA damage response–targeting therapies [22,23,24].

*CCNE1* amplification emerged as the most frequent gene gain event, affecting 13% of patients, and showed limited association with HRD positivity, consistent with previous genomic studies. This observation reinforces the recognition of *CCNE1*-amplified tumors as an HRD-independent subtype with limited expected PARP inhibitor sensitivity [12,13,14].

Pathogenic *BRCA* CNAs identified in tumor tissue strongly correlated with genomic instability (GI), with approximately 77% of CNA-positive tumors being GI-positive. Importantly, a substantial subset of these GI-positive, CNA-positive tumors retained wild-type *BRCA* sequences with no pathogenic SNVs or *indels*. This observation underscores that GI and HRD phenotypes can manifest even in the absence of canonical *BRCA* sequence mutations, likely driven by structural variants and alterations in other HRR genes. Pan-cancer genomic profiling studies have shown that a significant fraction of tumors exhibiting HRD signatures do not harbor sequence-based *BRCA* mutations, highlighting diverse mechanisms underlying the HRD phenotype beyond simple SNVs/indels of *BRCA* [11,25]. Similarly, analyses of HRD in tumors with non-*BRCA* HRR gene variations have documented elevated HRD scores in *BRCA* WT settings, suggesting that alterations in other repair genes contribute to GI and therapeutic vulnerabilities [25].

These results support the biological relevance of CNAs in the context of HRD and highlight the value of integrating somatic CNA analysis with SNV/*indel* assessment in tumor tissue. The exon-level resolution of the assay may provide additional granularity for the interpretation of structural variants within *BRCA* genes and other HRR-related genes. From a clinical perspective, these findings should be interpreted in light of previously published evidence suggesting a potential association between HRD-related genomic alterations and response to platinum-based chemotherapy or PARP inhibitors, including data from the PAOLA-1 trial [26]. We acknowledge that the absence of these additional data represents a limitation of the present study. However, a systematic evaluation of clinical data and related therapeutic implications, while highly relevant, is beyond the scope of the present study, which primarily focuses on technical and methodological aspects and will be addressed in dedicated future investigations.

Finally, high-resolution CNA detection in tumor tissue enables accurate identification of both somatic and germline *BRCA* LGRs, supporting a tumor-first testing strategy that effectively bridges precision oncology and hereditary cancer risk assessment. This strategy facilitates timely therapeutic decision-making while guiding confirmatory germline testing and genetic counseling when appropriate.

## 4. Materials and Methods

### 4.1. Patient Cohort

We retrospectively analyzed 760 samples of OC patients treated at Fondazione Policlinico Universitario Agostino Gemelli IRCCS from late 2022 to 2024. Formalin-Fixed, Paraffin-Embedded (FFPE) tumor tissues and matched peripheral blood were collected when available, within the framework of the institutional FPG500 program (ID: FPG500; Ethical Committee approval no. 3837), currently implemented at our Institution [27,28].

Inclusion criteria were a histologically confirmed diagnosis of OC, and in eligible samples, *BRCA* testing was performed using a commercial HRD assay. Patients with incomplete data or inadequate samples were excluded.

Clinical and pathological data, including age at diagnosis, histology, and disease stage, were extracted from medical records.

### 4.2. Sample Preparation and DNA Extraction and Qualification

FFPE specimens were obtained from the pathology department and selected based on predefined pre-analytical criteria assessed on hematoxylin and eosin (H&E)-stained sections by a qualified pathologist prior to macrodissection and DNA extraction. Inclusion criteria required a tumor cellularity >30% and necrosis <10%. These thresholds are consistent with commonly applied quality requirements for somatic variant analysis in FFPE material and with the manufacturer’s specifications for the HRD assay. Samples not meeting these criteria were excluded from the study.

While a formal quantitative tumor purity estimation was not performed for each individual case, pathologist-estimated tumor cellularity was used as the primary proxy for tumor content in the pre-analytical workflow. DNA was extracted using the MagCore^®^ Genomic DNA FFPE One-Step Kit (RBC Bioscience, Brussels, Belgium) according to the manufacturer’s instructions. DNA concentration and quality were assessed using a Qubit 3.0 Fluorometer (Thermo Fisher Scientific, Waltham, MA, USA) and the Genomic DNA ScreenTape Assay on TapeStation systems (Agilent Technologies Inc., Waldbronn, Germany), ensuring that all samples met the required input quality criteria for downstream molecular analyses.

### 4.3. HRD Assay and CNA Detection

All samples were analyzed with SOPHiA DDM™ Dx Homologous Recombination Deficiency Solution (SOPHiA GENETICS, Rolle, Switzerland), which integrates a proprietary CNA detection algorithm able to identify gene amplifications, deletions and exon-level CNAs. The assay workflow included DNA fragmentation, library preparation, hybridization-based target-capture NGS on the Illumina Nextseq550^TM^ (Illumina, San Diego, CA, USA). Data analysis was conducted using the assay’s proprietary software (SOPHiA DDM software version 4 (Sophia Genetics SA, Rolle, Switzerland)), which provides CNA calls based on read depth and coverage metrics. The CNA module performs the analysis in three steps: (1) Coverage normalization and segmentation was performed in two parallel streams supporting gene-level and exon-level analysis. Raw per-region read counts were doubly normalized across all target regions and co-processed samples; normalized coverage was averaged per gene to yield a gene-level coverage signal reflecting relative copy number, incorporating tumor cell fraction and sample ploidy. For exon-level analysis, the same normalization was applied independently within each gene, and the resulting intra-gene coverage signal was segmented using a Hidden Markov Model (HMM) to identify breakpoints between regions of constant copy number. Samples or genes with aberrant coverage profiles were flagged and excluded prior to calling. Before CNV calling, each sample was evaluated against predefined quality thresholds covering mapped fragment count, normalization outcome, and residual noise at both gene and exon levels. Samples passing all criteria underwent full combined gene- and exon-level analysis; those meeting only gene-level quality criteria were restricted to amplification calling; those failing remaining criteria were rejected. CNV calling applied distinct logic by gene class. For oncogenes, gain was assessed at the gene level with a distinction between low-level gain and high-level amplification. For all other genes, copy number status was assigned by thresholding the normalized coverage signal into discrete categories—gain, normal, suspected loss, and loss—in the absence of intra-gene breakpoints. When breakpoints were detected, gene- and exon-level signals were integrated per segment, with whole-gene calls made only when all segments met the same threshold. Exon-level segments were classified as exon gain, exon loss, suspected exon loss, or uncharacterized based on observed coverage relative to the diploid baseline; complex multi-segment patterns were reported as complex rearrangement. Full description of the method is available on the official SOPHiA GENETICS website (https://www.sophiagenetics.com/support, accessed on 1 August 2025).

*BRCA* full-gene and intragenic CNAs were included in the study if loss and suspected loss calls reached a value <1.2 or any gain >3.9.

Events were classified as pathogenic if previously reported in the literature or, if novel, if they involved critical domains of BRCA1 or BRCA2 with predicted impairment of gene function. Furthermore, a more restricted cut-off has been applied for gene gain (>4.5 copies). Additional categories, including those that could not be fully characterized, as well as those uncharacterized and complex rearrangements, were carefully reviewed and selected. Undefined rearrangement would be expected to be pathogenic, or in cases where multiple events were present within the gene with at least one pathogenic event (e.g., deletion and duplication). Intragenic duplications were classified as pathogenic if the location was predicted to disrupt the reading frame or critical domains of BRCA1 or BRCA2, negatively impacting protein production and/or function.

### 4.4. Orthogonal Validation of Somatic and Germline CNAs

The aim of this study was to evaluate the real-world clinical performance of the integrated CNA detection module within a commercially available, laboratory-implementable HRD assay applied in our diagnostic workflow. Orthogonal validation was performed using assays routinely used in accredited clinical laboratories for BRCA CNA detection, namely Multiplex Ligation-dependent Probe Amplification (MLPA; MRC Holland) and the amplicon-based Devyser BRCA NGS kit (Devyser, Stockholm, Sweden), both established reference methodologies in clinical molecular pathology.

Tumor-based CNA calls generated by the SOPHiA DDM™ Dx HRD Solution were confirmed in a subset of samples with putative BRCA alterations using the Devyser BRCA NGS kit, optimized for FFPE-derived DNA and enabling exon-level resolution of copy number alterations in BRCA genes [4,29].

To determine the germline or somatic origin of BRCA CNAs, matched peripheral blood DNA was analyzed within the same diagnostic workflow. Germline CNAs were assessed using validated assays according to routine laboratory practice and sample availability, including MLPA (MRC Holland), SOPHiA DDM™ Dx Hereditary Cancer Solution (SOPHiA GENETICS, Switzerland), or the Devyser BRCA NGS kit [4,29,30].

## 5. Conclusions

The ability to detect *BRCA* CNAs directly from tumor tissue may improve diagnostic workflows. In this context, tumor-based CNA detection may help guide the selection of cases for reflex germline MLPA testing, with germline confirmation focused on patients with suspected germline variants or CNAs.

Moreover, since HRD status is defined as positive in the presence of a *BRCA* alteration regardless of the GI score [31], a comprehensive assessment of *BRCA* status is essential. This requires the identification of all clinically relevant types of *BRCA* alterations, including SNVs, *indels*, and CNAs.

Incorporating high-resolution CNA detection into routine HRD workflows may improve the sensitivity of genomic profiling by enabling a more detailed characterization of somatic alterations, including copy number events affecting *BRCA* genes. This integrated approach has the potential to refine molecular stratification in ovarian cancer, support more comprehensive tumor profiling within established testing algorithms, and potentially expand the population of patients who may benefit from PARP inhibitors and other therapies targeting genomic instability.

Collectively, our findings reinforce the value of integrating CNA analysis into combined somatic–germline workflows, providing a more comprehensive framework for precision oncology and optimized management of patients with OC.

## Figures and Tables

**Table 1:** Summary of CNA-related events in *BRCA* genes.

Step	Number of Events	Percentage (%)
Total events	1592	/
Rejected (QC failure)	28	1.8
Assessed events	1564	98.2
Normal (no CNAs)	1509	96.5 *
CNA-positive (gain/loss)	55	3.5 *

* Percentages for “Normal” and “CNA-positive” are calculated using the number of assessed events (*n* = 1564) as the denominator. “Rejected” and “Assessed events” are calculated using total events (*n* = 1592) as the denominator.

**Table 2:** Summary of *BRCA* CNA status in OC cohort.

Total Patients	WT	Pathogenic CNA (Including Confirmed Germline Origin)	Pathogenic CNA (Excluding Confirmed Germline Origin)
759	721	38	28
	95%	5%	3.7%
*BRCA1*	/	26 (68%)	16 (57%)
*BRCA2*	/	12 (32%)	12 (43%)
GI+		26 (77%) *	
GI−		8 (23%) *	

* Percentage values obtained by excluding 4 samples with GI not defined or rejected (total = 34).

**Table 3:** Full list of pathogenic/likely pathogenic (*) CNA events in *BRCA* genes.

Case	Gene	Gene-Level Status	Exon-Level Status	Segment Regions	Segment Coverage Level	Genomic Integrity STATUS (**)	BRCA Status (**)
1	*BRCA2*	Exon-level event	Exon loss	ex19–ex21	0.8	1	0
2	*BRCA1*	Suspected loss	Normal	ex24–ex2	1.2	1	1
3	*BRCA1*	Exon-level event	Exon gain	ex2	4.7	1	1
4	*BRCA1*	Suspected loss	Normal	ex24–ex2	1.1	0	0
5	*BRCA1*	Exon-level event	Exon loss	ex23	0.8	1	0
6	*BRCA1*	Suspected loss	Normal	ex24–ex2	1	0	0
7	*BRCA2*	Suspected loss	Normal	ex2–ex27	1	1	0
8	*BRCA1*	Exon-level event	Exon loss	ex22–ex21	0.6	1	0
9	*BRCA2*	Loss	Normal	ex2–ex27	0.5	1	0
10	*BRCA2*	Exon-level event	Suspected exon loss	ex13	1.2	1	1
11	*BRCA1*	Exon-level event	Exon loss	ex19–ex18	0.6	1	0
12	*BRCA1*	Loss	Normal	ex24–ex2	0.6	1	0
13	*BRCA1*	Exon-level event	Exon loss	ex19–ex18	0.7	ND	0
14	*BRCA1*	Exon-level event	Exon loss	ex23	0.4	1	0
15	*BRCA2*	Exon-level event	Exon loss	ex26–ex27	0.6	ND	0
16	*BRCA2*	Suspected loss	Normal	ex2–ex27	1	0	0
17	*BRCA2*	Exon-level event	Uncharacterized	ex2–ex18	1.3	1	0
18	*BRCA1*	Suspected loss	Normal	ex24–ex2	1.1	0	0
19	*BRCA2*	Suspected loss	Normal	ex2–ex27	1.1	0	0
20	*BRCA1*	Exon-level event	Exon loss	ex17–ex16	0.5	1	0
21	*BRCA1*	Exon-level event	Exon gain	ex2	3.9	ND	0
22	*BRCA2*	Suspected loss	Normal	ex2–ex27	1	1	1
23	*BRCA2*	Suspected loss	Normal	ex2–ex27	0.9	1	0
24	*BRCA1*	Exon-level event	Exon loss	ex2	0.5	1	0
25	*BRCA1*	Exon-level event	Exon loss	ex8–ex11	0.7	1	0
26	*BRCA1*	Exon-level event	Exon loss	ex20	0.4	0	0
27	*BRCA1*	Exon-level event	Exon loss	ex20	0.2	1	0
28	*BRCA1*	Exon-level event	Exon loss	ex2	0.5	1	0
29	*BRCA2*	Exon-level event	Complex rearrangement	ex4–ex27	<1	1	0
30	*BRCA1*	Exon-level event	Exon loss	ex17–ex16	0.2	1	0
31	*BRCA1*	Exon-level event	Uncharacterized	ex15–ex2	3.1	0	0
32	*BRCA2*	Gain	Normal	ex2–ex27	4.5	0	0
33	*BRCA1*	Exon-level event	Exon loss	ex2	0.5	1	ND
34	*BRCA1*	Exon-level event	Exon loss	ex19	0.7	1	ND
35	*BRCA1*	Exon-level event	Exon loss	ex17–ex16	0.5	1	0
36	*BRCA1*	Exon-level event	Exon loss	ex7–ex4	0.3	1	ND
37	*BRCA1*	Exon-level event	Exon loss	ex3–ex2	0.5	ND	0
38	*BRCA1*	Exon-level event	Suspected exon loss	ex16	1	1	0

(*) Full list of pathogenic/likely pathogenic CNA events detected by the CNA algorithm. Gene loss and suspected loss with a value <1.2 or any gene gain >4.5 were considered in this dataset, as well as exon loss and suspected loss with a value <1.2 or any exon gain >3.9. Uncharacterized and complex rearrangement samples have been carefully reviewed and selected. (**) Note: Genomic integrity status: 0 = negative, 1 = positive; BRCA: 0 = wild-type (WT), 1 = positive.

**Table 4:** Correlation of GI and *BRCA* status in OC patients found with pathogenic CNAs.

GI Status	Total Cases (*n*)	* *BRCA* WT	* *BRCA* Pathogenic	* *BRCA* VUS
GI+	26	19 (73.1%)	4 (15.4%)	3 (11.5%)
GI−	8	8 (100%)	0 (0%)	0 (0%)

* BRCA WT/Pathogenic/VUS refers to SNVs and small indels.

**Table 5:** Correlation between HRD status and *CCNE1* amplification.

Amplification Level	Total Cases *	HRD+	HRD−	NA *
Mild amplification (3.3–5.9)	59	21 (36%)	38 (64%)	2
High amplification ≥ 6	37	(11%)	33 (89%)	1

* Not applicable (NA).

## Data Availability

The original contributions presented in this study are included in the article. Further inquiries can be directed to the corresponding author.
